# Evidence of genetic overlap and causal relationships between blood-based biochemical traits and human cortical anatomy

**DOI:** 10.1038/s41398-022-02141-3

**Published:** 2022-09-08

**Authors:** Dylan J. Kiltschewskij, William R. Reay, Murray J. Cairns

**Affiliations:** 1 School of Biomedical Sciences and Pharmacy, The University of Newcastle, Callaghan, NSW Australia; 2 Precision Medicine Research Program, Hunter Medical Research Institute, New Lambton, NSW Australia

**Keywords:** Genomics, Predictive markers, Molecular neuroscience

## Abstract

Psychiatric disorders such as schizophrenia are commonly associated with structural brain alterations affecting the cortex. Recent genetic evidence suggests circulating metabolites and other biochemical traits play a causal role in many psychiatric disorders which could be mediated by changes in the cerebral cortex. Here, we leveraged publicly available genome-wide association study data to explore shared genetic architecture and evidence for causal relationships between a panel of 50 biochemical traits and measures of cortical thickness and surface area. Linkage disequilibrium score regression identified 191 genetically correlated biochemical-cortical trait pairings, with consistent representation of blood cell counts and other biomarkers such as C-reactive protein (CRP), haemoglobin and calcium. Spatially organised patterns of genetic correlation were additionally uncovered upon clustering of region-specific correlation profiles. Interestingly, by employing latent causal variable models, we found strong evidence suggesting CRP and vitamin D exert causal effects on region-specific cortical thickness, with univariable and multivariable Mendelian randomization further supporting a negative causal relationship between serum CRP levels and thickness of the lingual region. Our findings suggest a subset of biochemical traits exhibit shared genetic architecture and potentially causal relationships with cortical structure in functionally distinct regions, which may contribute to alteration of cortical structure in psychiatric disorders.

## Introduction

The pathogenesis of psychiatric disorders is underpinned by a complex interplay of genetic and environmental risk factors. Large-scale genetic studies have identified a strong genetic component of these disorders, characterised by a vast polygenic burden across the genome arising from variants ranging in frequency from common to ultra-rare, as well as large effect size structural variants [1, 2]. Cross-disorder analyses have additionally revealed substantial proportions of shared genetic architecture between psychiatric disorders [3–5], potentially contributing to similarities in clinical presentation and the frequency of observed psychiatric comorbidities. In conjunction with shared genetic risk, many psychiatric disorders are also associated with alteration of brain structure which may account for a range of psychiatric symptoms and offer utility in dissecting underlying pathophysiological mechanisms. Widespread dysregulation of cortical structures in particular has emerged as a consistent feature, with recent neuroanatomical meta-analyses observing grey matter reductions in overlapping cortical regions for schizophrenia, major depressive disorder, bipolar disorder and obsessive-compulsive disorder, amongst others, while structural alterations unique to each disorder have also been identified [6–8]. Although further investigation of these neuroanatomical changes may assist in the discovery of clinically actionable pathways and biomarkers, the fundamental genetic and environmental factors associated with alterations to brain structure remain poorly understood.

Dysregulation of circulating biochemical factors is one broad mechanism through which variations in cortical structure may arise in psychiatric and neurodegenerative disorders. During the development of the central nervous system, alteration of these circulating factors could plausibly interfere with patterns of neuronal differentiation and migration important for cortical development, while dysregulation in the adult brain may disrupt neuronal cytoarchitecture and integrity. Systemic effects have been widely studied in psychiatry, as many of these biochemical variables can be modulated through existing drugs or lifestyle intervention, potentially informing novel treatment interventions. For example, observational evidence suggests elevated C-reactive protein (CRP) in the serum of individuals with schizophrenia is associated with cortical thinning in the frontal, insula and temporal regions [9]. An array of studies has identified many other blood-based biochemical traits with potential diagnostic or prognostic utility [10, 11], however a major drawback of these observational studies is their inability to discriminate between correlations and causal relationships, thus any biological effects resulting in alteration of brain structure are difficult to detect. To this end, genome-wide association studies (GWAS) are proving increasingly valuable for examining and distinguishing genetic correlations and genetically informed causal relationships amongst traits of interest. In particular, germline genetic variants are largely fixed at birth which helps to mitigate effects due to reverse causation in observational studies [12]. Indeed, recent studies utilising GWAS-guided methods of causal inference such as Mendelian randomization have uncovered putative causal relationships between blood-based biochemical traits and psychiatric disorders, using genome-wide significant SNPs as genetic proxies for biochemical exposures [13, 14]. However, no studies to date have examined the fundamental relationship between biochemical traits and structure of the human cerebral cortex utilising population-level genetic information. To address this, we employed GWAS summary statistics for a diverse panel of 50 blood-based biochemical traits (UK biobank; http://www.nealelab.is/uk-biobank) to examine shared genetic architecture and putative causal relationships with cortical thickness and surface area measurements (ENIGMA [15]). Our analyses revealed a diverse range of genetic correlations and identified clusters of cortical regions and biochemical traits with similar correlation profiles. In addition, we uncovered evidence for region-specific causal relationships, suggesting subsets of circulating biochemical traits may exert biologically significant effects on cortical structure with direct relevance to psychiatric disorders.

## Materials and methods

### Genome-wide association study data

GWAS summary statistics for cortical surface area (SA) and thickness (TH) as measured via magnetic resonance imaging were obtained from the ENIGMA consortium study [15]. These data were generated via meta-analysis of 33,992 individuals of European ancestry, sourced from 49 independent cohorts in the ENIGMA consortium (23,909 individuals) as well as the UK Biobank (UKBB; 10,083 individuals) [16]. We utilised the global measures of mean cortical TH and total SA, as well as regional measures for 34 distinct cortical areas as defined by the Desikan-Killiany atlas. All summary statistics were covaried for age, age^2^, sex, sex-by-age and age^2^ interactions, ancestry (first four multidimensional scaling components), diagnostic status (for case vs control studies) and dummy variables correcting for the use of multiple scanners. Furthermore, we note that our analyses utilised the most recent release of the ENIGMA summary statistics, in which a previous error affecting regional cortical thickness measures has been corrected. All data are available upon request at http://enigma.ini.usc.edu/research/download-enigma-gwas-results/. See Table S1 for further details.

We additionally obtained summary statistics for a series of blood-based biochemical traits, uniformly produced from a large cohort of >300,000 individuals in the UK biobank (available from http://www.nealelab.is/uk-biobank; round 2). Biochemical traits with high or medium confidence SNP heritability estimates significantly different from zero were utilised, yielding a total of 50 traits including blood cell counts, metabolites, enzymes, lipids and other biomarkers, all of which were inverse rank normal transformed. See Table S1 for further details.

For Mendelian randomization, non-UKBB biochemical GWAS were used to eliminate statistical inflation arising from sample overlap between the ENIGMA and UKBB cohorts. Specifically, non-UKBB GWAS for CRP (ieu-b-35) [17], vitamin D (ebi-a-GCST005367) [18], body mass index (BMI; ieu-a-835) [19], interleukin-6/interleukin-6 receptor (IL-6/IL-6R; prot-b-2/prot-b-23) [20] and fasting glucose (ieu-b-113 & ieu-b-114) [21, 22] were sourced from https://gwas.mrcieu.ac.uk. Red blood cell (RBC) count (GCST004601) and haematocrit percentage (GCST004604) [23] were downloaded from GWAS Catalog (https://ebi.ac.uk/).

### Genetic correlation

Genetic correlations amongst cortical and biochemical traits were examined via linkage disequilibrium score regression (LDSR) using the *ldsc* package [24], as described in detail previously [25]. All summary statistics were converted to a standardised “munged” format, wherein approximately one million HapMap 3 SNPs outside the major histocompatibility complex with minor allele frequency > 0.05 were retained for further analysis. We utilised linkage disequilibrium (LD) scores (“eur_w_ld_chr”) previously computed from the 1000 Genomes Project European reference panel available at https://alkesgroup.broadinstitute.org/LDSCORE/. Correlations with Bonferroni-corrected *P* < 0.05 (*P*_*Global*_ = 5 × 10^–4^, 100 comparisons; *P*_*Regional*_ = 1.471 × 10^–5^, 3,400 comparisons) were considered statistically significant, while a Benjamini-Hochberg false discovery rate (FDR) < 0.05 was considered suggestively significant. LDSR *Z*-scores were subjected to unsupervised clustering by finite Gaussian mixture modelling (GMM) using the mclust R package (v5.4.7) [26], with the number of components (clusters) selected using the largest Bayesian Information Criterion (BIC) value.

### Latent causal variable models

A latent causal variable (LCV) model was used to estimate genetic causality as described previously [27]. Briefly, the LCV model tests for partial genetic causality by comparing the mixed fourth moments (cokurtosis) of SNP marginal effect size distributions for each trait to determine whether SNPs affecting trait 1 have proportional effects on trait 2, but not vice versa. The LCV model reports a posterior mean genetic causality proportion ($\widehat {GCP}$) of trait 1 on trait 2, wherein $\widehat {GCP}$ = 0 suggest no causal relationship, $\widehat {GCP}$ > 0 implies trait 1 is partially genetically causal for trait 2 and $\widehat {GCP}$ < 0 suggests trait 2 is partially genetically causal for trait 1. We consider significant |$\widehat {GCP}$| estimates ≥ 0.6 as strong evidence for partial genetic causality as shown previously [27], noting that $\widehat {GCP}$ summarises the strength of evidence for a genetically causal relationship, rather than the magnitude of a causal effect. All summary statistics subjected to LCV modelling were “munged” prior to analysis as recommended.

### Mendelian randomisation

Two-sample Mendelian randomization (MR) was employed to further explore and quantify the magnitude of causal relationships amongst trait pairings with evidence for partial genetic causality. MR leverages genetic instrumental variables (IVs) rigorously associated with an exposure – most commonly independent genome-wide significant SNPs – to estimate the causal effect of the exposure on an outcome. We specifically tested the effect of biochemical traits on structural cortical measurements utilizing LD-clumped, non-palindromic IVs sourced from well-powered non-UK Biobank GWAS to eliminate statistical inflation arising from sample overlap between the ENIGMA and UKBB cohorts. For all biochemical GWAS, the IV *F*-statistic was >10, indicating IVs for these traits are sufficiently powered [28].

All exposure-outcome trait pairings were analysed via five MR models with differing underlying assumptions regarding the validity of using SNPs as IVs. The inverse variance weighted model with multiplicative random effects (IVW_m_) was used as our principal model, which is generally considered the most-well powered model but has a zero percent breakdown point, and thus, assumes all IVs are valid. Furthermore, IVW_m_ also assumes there is some pleiotropy amongst the IVs, but the average pleiotropic effect is zero [29]. IVW_m_ *P* values were adjusted via Bonferroni method and the Benjamini-Hochberg FDR. We also employed four supplementary methods for the purposes of sensitivity analysis, for which the directions and magnitudes of their estimates were compared to the IVW_m_ method. Specifically, we included an IVW estimator with fixed effects (IVW_f_) which specifically assumes zero pleiotropy, as well as a weighted median model [30], a weighted mode model [31] and a MR Egger model [32]. We also assessed whether there was further evidence of horizontal pleiotropy or other outlier effects via the following: Cochran’s *Q* to test heterogeneity in the individual IV exposure-outcome estimates [33], iteratively leaving out each IV and recalculating the IVW estimate (leave-one-out) [33], and the MR PRESSO test of global pleiotropy, which is also related to heterogeneity [34]. Finally, the Steiger directionality test was implemented to evaluate evidence that the assumed causal direction (biochemical trait → cortical property) was correct through comparing the variance explained by the IVs in the exposure to their association with the cortical outcome GWAS [35].

Since circulating CRP biology is thought to be closely associated with BMI and interleukin-6 (IL-6) signalling, both upstream and downstream of its synthesis [19, 20], we estimated the direct effect of CRP on regional cortical thickness conditioned on these variables using multivariable MR (MVMR) models. MVMR leverages genome-wide significant SNPs associated with at least one exposure as IVs, with the IV effect on all exposures considered. A total of three separate multivariable models were tested: CRP conditioned on BMI, CRP conditioned on IL-6 and the IL-6 receptor (IL-6R), and CRP conditioned on BMI and IL-6R. We note that for the CRP, IL-6 and IL6-R model, an *F* statistic > 10 was not achieved for IL-6 and IL-6R, however, we included this analysis noting that MVMR with weak instruments is likely to bias the causal estimate towards zero [36]. Four MVMR methods were implemented: an IVW MVMR estimator with multiplicative random effects, a median based MVMR estimator, an Egger regression-based MVMR estimator, and a LASSO-type regularisation approach in which intercepts are shrunk towards zero for valid IVs [37]. All MR analyses were conducted using the *TwoSampleMR* (v.0.5.5) [38], *MRPRESSO* (v.1.0) [34] and *MVMR* (v.0.3) [36] *R* packages.

### Transcriptome-wide association studies

Transcriptome-wide association studies (TWAS) were conducted using the *FUSION* approach to identify genes associated with biochemical traits and cortical TH, as well as a predicted direction of expression [39]. TWAS utilizes *cis*-acting genetic variants to predict gene expression optimised through competing multivariate models (genetically regulated expression – GReX). Genes that have significantly heritable expression explained by *cis*-acting variants can be evaluated with this method. Specifically, the most predictive GReX model is utilised to correlate predicted expression with the GWAS trait of interest by integrating the SNP weights from the expression model with their corresponding effect estimated by the GWAS. As a result, the covariance between expression and the trait can be leveraged to interpret the direction of association positively correlated with the trait – for example, a gene with a positive TWAS test statistic (*Z* > 0) in the context of a continuous GWAS trait signifies that increased expression of the gene is correlated with an increase in the trait. We utilised SNP weights from both the blood and cortex from GTEx v7 [40]. Since CRP has a well-documented function in the liver, we also included liver expression weights for this exposure. Tissue-specific, transcriptome-wide genetic correlations between traits were additionally examined using the *RHOGE R* package [41].

## Results

### Extensive genetic correlation among biochemical traits and cortical properties

**Fig. 1:**
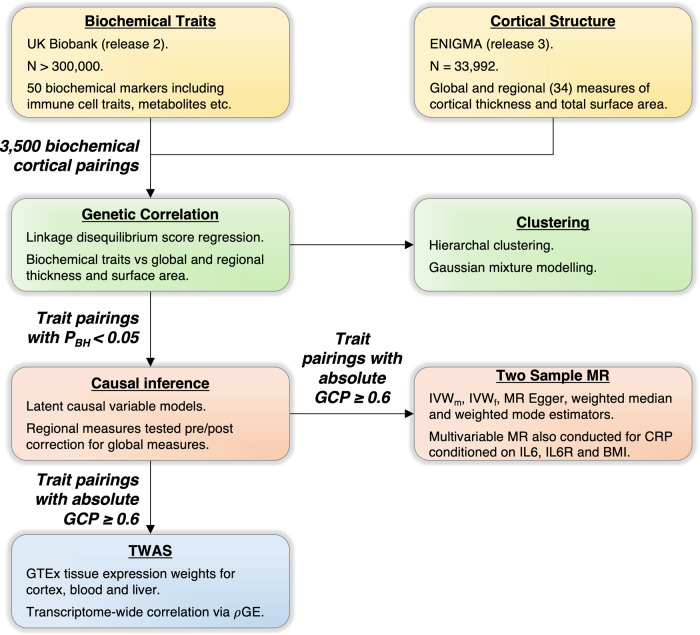
Genetic investigation of correlation and causation amongst biochemical traits and cortical structure. GWAS summary statistics for 50 biochemical traits and measures of cortical thickness and surface area were obtained from the UK Biobank and ENIGMA consortium, respectively. Genetic correlation amongst all biochemical-cortical trait pairings was analysed via linkage disequilibrium score regression, and clustering of correlation profiles was examined via hierarchal clustering and gaussian mixture modelling. All trait pairings with suggestive genetic correlation (*P*_*Benjamini-Hochberg*_ < 0.05) were subjected to causal inference using latent causal variable models. Pairings with an absolute genetic causality proportion (GCP) ≥ 0.6 were further subjected to two-sample Mendelian randomization (MR) to obtain causal estimates, as well as transcriptome-wide association studies (TWAS) to examine shared associated genes.

We estimated genetic correlation between a panel of blood-based biochemical traits with respect to mean cortical thickness (TH) and total surface area (SA) using LDSR (see Fig. 1 for an overview of the cohorts and methods employed in this study). This panel was composed of all available biochemical traits measured in the UKBB with high or medium confidence SNP heritability estimates (50 in total), including blood cell counts, enzymes, lipids and vitamins, amongst others. We assert this hypothesis-free method enables systematic and unbiased identification of diverse biochemical traits genetically correlated with cortical structure, in a manner which benefits from utilizing the large, uniformly processed UKBB cohort.

**Fig. 2:**
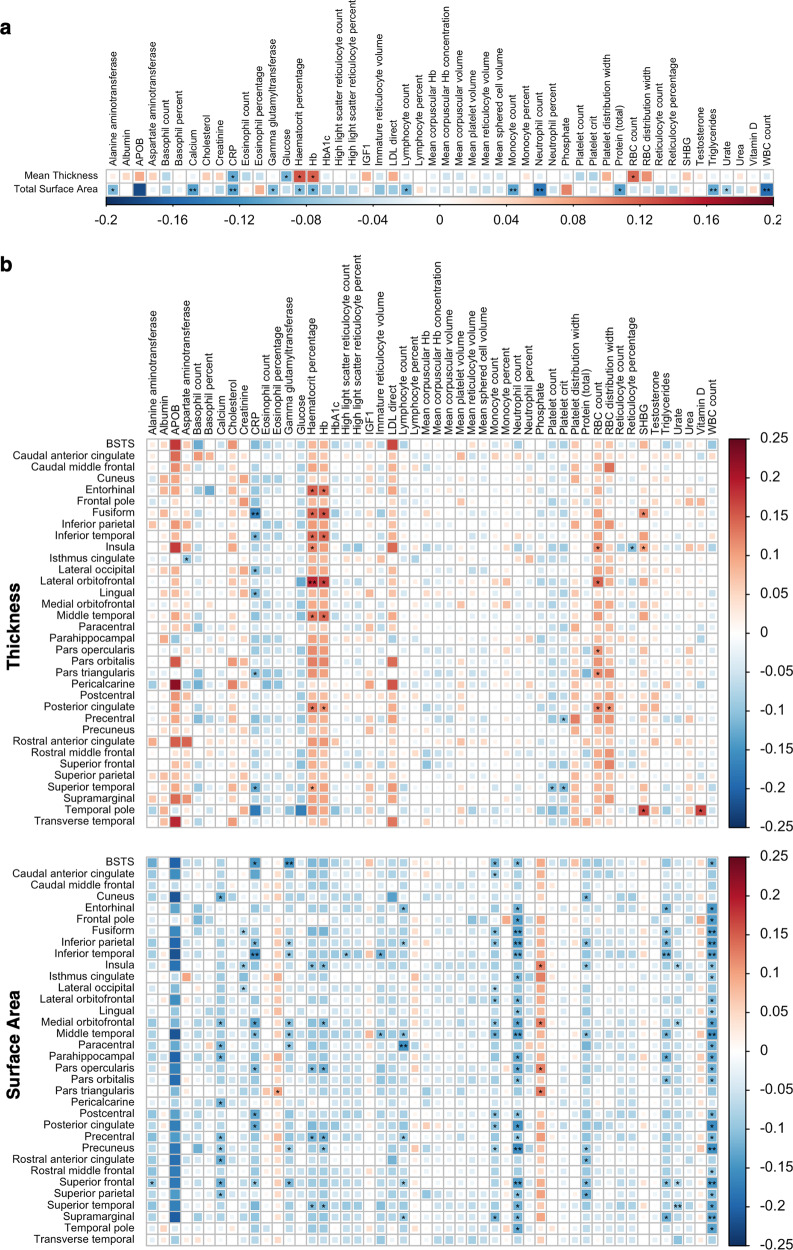
Genetic correlation amongst GWAS for biochemical and cortical structural traits. **a** Heatmap of LDSR genetic correlation coefficients (*r*_*g*_) between mean cortical TH and total cortical SA with respect to 50 biochemical traits. *P_*Benjamini-Hochberg*_ < 0.05, **P_*Bonferroni*_ < 0.05. **b** As in **a**, except depicting genetic correlations between biochemical traits with respect to TH (top) and SA (bottom) of 34 cortical regions (averaged across both hemispheres) as defined by the Desikan-Killiany atlas. BSTS = banks of the superior temporal sulcus.

With respect to total cortical SA, 13 negatively correlated biochemical traits (six *P*_*Bonferroni*_ < 0.05, seven *P*_*BH*_ < 0.05) were identified, including blood cell counts (white blood cells (WBCs), neutrophils, monocytes, lymphocytes and haematocrit percentage), nutrients (calcium, triglycerides and protein (total)) and enzymes (gamma-glutamyltransferase and alanine aminotransferase), as well as CRP, urate and haemoglobin (Fig. 2a, Table S2). For mean cortical TH, five biochemical traits were genetically correlated (all *P*_*BH*_ < 0.05). Specifically, haematocrit percentage (*r*_*g*_ = 0.125, *SE* = 0.037, *P* = 0.0008), haemoglobin (*r*_*g*_ = 0.12, *SE* = 0.037, *P* = 0.0011) and RBC count (*r*_*g*_ = 0.117, *SE* = 0.035, *P* = 0.0009) were positively correlated, while CRP (*r*_*g*_ = –0.103, *SE* = 0.034, *P* = 0.0027) and glucose (*r*_*g*_ = –0.096, *SE* = 0.036, *P* = 0.0086) were negatively correlated (Fig. 2a, Table S2). Only CRP displayed a consistent correlation coefficient for total SA (*r*_*g*_ = –0.108, *SE* = 0.030, *P* = 0.0003) and mean TH, suggesting total cortical SA and mean TH exhibit divergent patterns of genetic correlation with respect to the tested biochemical traits.

We additionally explored site-specific partitioning of genetic correlations by analysing measures of SA and TH for 34 distinct regions. For regional SA, 138 correlated trait pairings (18 *P*_*Bonferroni*_ < 0.05, 120 *P*_*BH*_ < 0.05) were identified, involving 18 biochemical traits and 32 cortical regions (Fig. 2b, Table S3). A clear majority (92%) of these pairings involved biochemical traits which were also correlated with total SA – such as WBC count (26 correlations), neutrophil count (23) and monocyte count (12) – however a subset were exclusively correlated at the regional level, including phosphate (4), creatinine (3) and immature reticulocyte volume (2). Overall, the superior frontal (10), middle temporal (9), medial orbitofrontal (8) and inferior parietal (8) regions were most consistently associated. Genetic correlation for regional TH was comparatively modest, with 35 (two *P*_*Bonferroni*_ < 0.05, 33 *P*_*BH*_ < 0.05) correlated trait pairings involving 11 biochemical traits and 15 cortical phenotypes (Fig. 2b, Table S3). Again, a high proportion (71.4%) of associated biochemical traits were correlated with mean cortical TH, however traits such as sex hormone-binding globulin (3) and platelet crit (2) emerged with region specific correlations. We additionally identified the fusiform, insula, posterior cingulate and superior temporal areas (four correlations each) as most consistently represented. Strikingly, only the CRP and the inferior temporal pairing exhibited consistent correlation in the SA (*r*_*g*_ = –0.160, *SE* = 0.034, *P* = 3.39 × 10^−6^) and TH (*r*_*g*_ = –0.115, *SE* = 0.034, *P* = 0.0008) analyses.

### Clustering of genetic correlation profiles reveals cortical regions with similar biochemical relationships

**Fig. 3:**
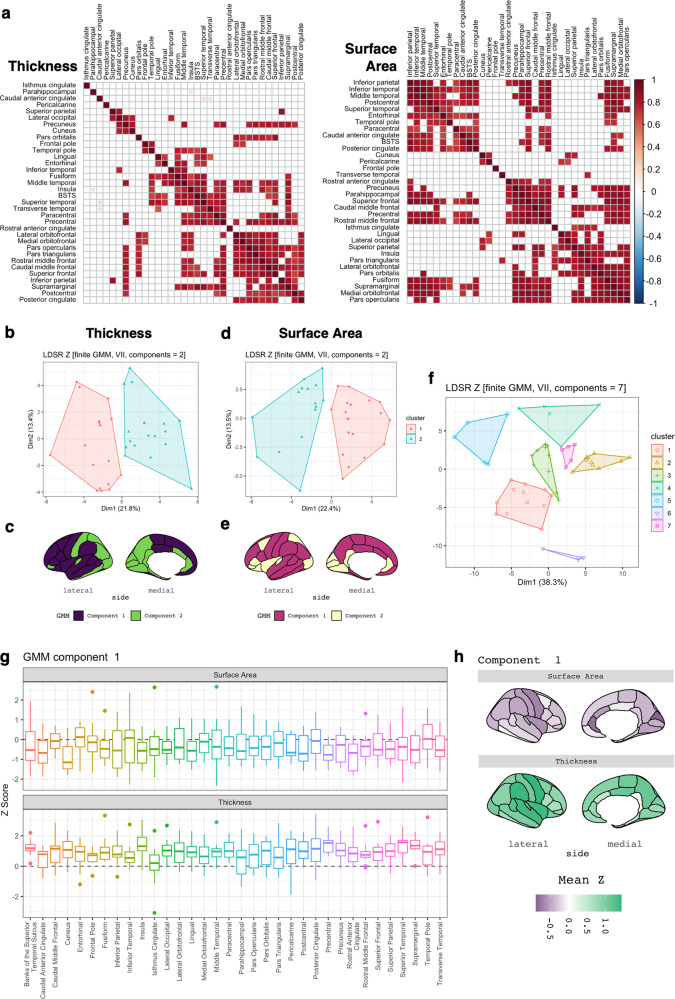
Clustering of cortical and biochemical correlation profiles. **a** Heatmaps depicting correlations between cortical regions after comparison of LDSR biochemical *Z-*score profiles. Cortical trait pairings with *P*_*Bonferroni*_ < 0.05 are shown, with hierarchal clustering via Ward’s D2 method. BSTS = banks of the superior temporal sulcus. **b** Clustering of cortical TH measures via biochemical LDSR *Z*-score profiles using finite Gaussian mixture modelling (GMM). Optimal parameterisation of the covariance matrix was obtained using two clusters (components) of cortical regions with spherical distribution, variable volume and equal shape (VII). Components are plotted via their contribution to the first and second principal components of the LDSR *Z* matrix. **c** Spatial distribution of GMM components identified in panel (**b**). Note that component one (purple) is largely confined to the frontal and temporal lobes, whereas component two predominantly consists of parietal and occipital regions. **d** & **e** As in **b** & **c**, except examining clusters of cortical regions subset by SA. **f** GMM clustering of biochemical traits by their TH and SA *Z-*score profiles, which revealed a total of seven biochemical clusters. **g** Boxplots of LDSR *Z*-scores for all biochemical traits in component one, including *APOB*, *albumin*, *aspartate aminotransferase*, *cholesterol*, *IGF1*, *LDL direct*, *monocyte percent*, *platelet distribution width*, *RBC distribution width*, *sex hormone binding globulin*, *testosterone* and *urea*. Note that *Z*-scores for these biochemical traits were generally negative with respect to cortical SA and positive with respect to cortical TH. **h** Spatial organisation of mean *Z-*scores for biochemical traits in component 1.

Hierarchal clustering of cortical regions via their biochemical correlation profiles revealed many positively correlated regions which loosely clustered by their spatial proximity, while physiologically related biochemical traits (e.g. immune cell traits, reticulocyte traits) tended to cluster in both the SA and TH analyses (Fig. 3a & Fig. S1). For instance, analysis of cortical TH revealed a subset of seven adjacent regions in the frontal lobe (lateral and medial orbitofrontal, pars opercularis, pars triangularis, rostral and caudal middle frontal and superior frontal) with highly correlated biochemical *Z*-score profiles (Fig. 3a). We subsequently employed finite Gaussian mixture modelling (GMM) to identify latent genetic relationships between cortical measures and biochemical traits. Two distinct components (clusters) of cortical regions with respect to their biochemical correlations were identified in the TH analysis, with cluster 1 (17 regions) largely localised to the frontal and temporal lobes, while cluster 2 (17 regions) was predominantly parietal and occipital (Fig. 3b, c, Table S4). Although two clusters were also observed in the SA analysis, the spatial distribution was less clear, as cluster 1 (19 regions) contained a series of contiguous regions spanning multiple cortical lobes, whereas cluster 2 (15 regions) consisted of regions with relatively diffuse spatial localisation (Fig. 3d, e, Table S5). GMM of the biochemical traits identified seven clusters with similar SA and TH correlation profiles (Fig. 3f, Table S6). Clusters one and six were largely composed of traits with negative LDSR *Z-*scores for regional SA and positive scores for TH, including cholesterol, testosterone and RBC-related traits, amongst others (see Fig. 3g, h for representative cluster, see Fig. S2 for full data). Clusters two, three and seven were also negatively correlated with SA and exhibited discordant correlations with TH, with consistent representation of reticulocyte and immune cell traits. Notably, cluster four was negatively correlated with both measures and included CRP, glucose, glycated haemoglobin and platelet-related traits, while cluster five was positive for SA and negative for TH.

### Strong evidence for causal relationships between biochemical traits and regional cortical thickness

**Fig. 4:**
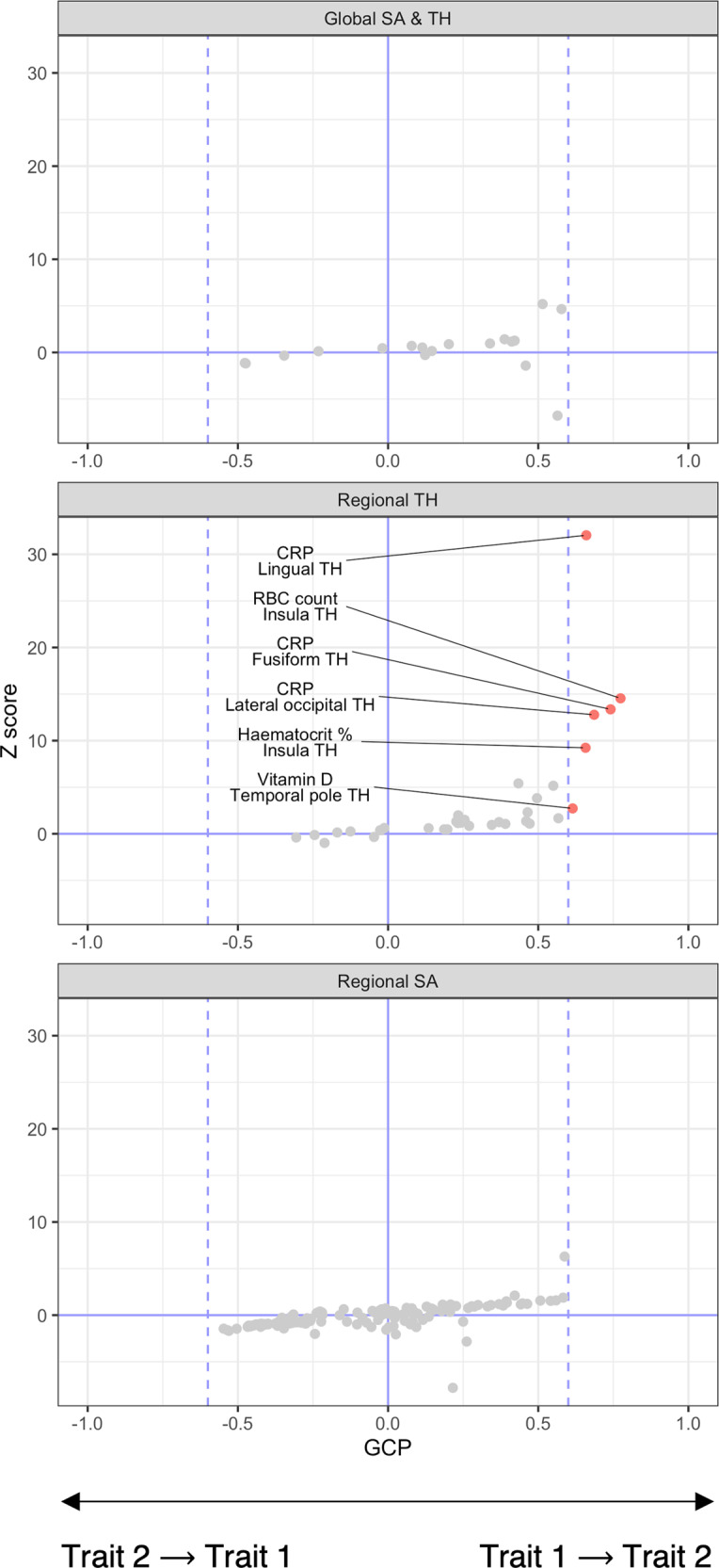
Genetically inferred causal relationships amongst biochemical and cortical traits. Biochemical-cortical trait pairings with suggestive (*P*_*BH*_ < 0.05) or significant (*P*_*Bonferroni*_ < 0.05) genetic correlation were subjected to latent causal variable (LCV) analysis. Of 35 tested trait pairings associated with regional cortical TH, six exhibited strong evidence (|$\widehat {GCP}$| > 0.6, dashed blue vertical line) for a causal relationship, with all involving a biochemical trait acting on the cortical trait. In contrast, no trait pairings associated with the global measures of total SA and mean TH, or regional SA exhibited strong evidence for causality.

We next employed a latent causal variable (LCV) model to examine evidence for partial genetic causality amongst all correlated trait pairings. For the mean cortical TH and total SA analyses, no trait pairings exhibited strong evidence for a putative causal relationship, with Glucose → mean TH ($\widehat {GCP}$ = 0.58, *SE* = 0.26, *P* = 1.01 × 10^−5^) and CRP → total SA ($\widehat {GCP}$ = 0.56, *SE* = 0.10, *P* = 8.59 × 10^−10^) marginally failing to exceed the mean posterior |$\widehat {GCP}|$ ≥ 0.6 threshold (Fig. 4, Table S7). We therefore constructed LCV models for all region-specific genetically correlated trait pairings, revealing a total of six pairings with strong evidence for partial genetic causality, including: CRP on fusiform TH ($\widehat {GCP}$ = 0.74, *SE* = 0.15, *P* = 6.94 × 10^−24^), lateral occipital TH ($\widehat {GCP}$ = 0.69, *SE* = 0.16, *P* = 1.15 × 10^−22^) and lingual TH ($\widehat {GCP}$ = 0.66, *SE* = 0.14, *P* = 4.55 × 10^−54^), RBC count on insula TH ($\widehat {GCP}$ = 0.77, *SE* = 0.17, *P* = 2.6 × 10^−26^), haematocrit percentage on insula TH ($\widehat {GCP}$ = 0.66, *SE* = 0.22, *P* = 5.14 × 10^−15^) and vitamin D on temporal pole TH ($\widehat {GCP}$ = 0.61, *SE* = 0.24, *P* = 0.0076; Fig. 4, Table 1, Table S8). Of these, only the vitamin D → temporal pole TH pairing did not survive correction for multiple testing or the FDR, however we retained this pairing for further analysis due to the strong $\widehat {GCP}$ estimate. To ensure these results were not affected by global measures, we repeated this analysis using GWAS summary statistics covaried for mean cortical TH. Strong evidence for partial genetic causality remained after this adjustment for CRP on lingual TH ($\widehat {GCP}$ = 0.69, *SE* = 0.19, *P* = 1.41 × 10^−73^) and lateral occipital TH ($\widehat {GCP}$ = 0.6, *SE* = 0.21, *P* = 5.93 × 10^−6^) and vitamin D on temporal pole TH ($\widehat {GCP}$ = 0.72, *SE* = 0.18, *P* = 2.63 × 10^−7^; Table 1). Considering the sign of genetic correlations between these traits, it is therefore likely that increased CRP levels are associated with decreased lingual (*r*_*g*_ = –0.118) and lateral occipital (*r*_*g*_ = –0.111) TH, whereas increased vitamin D is associated with increased temporal pole TH (*r*_*g*_ = 0.169). Evidence implicating the remaining three pairings was ablated after correction for mean cortical TH ($\widehat {GCP}$ ≤ 0.46, *SE* ≥ 0.34, *P* ≥ 0.25; Table 1).

**Table 1:** Latent casual variable analyses for trait pairings with significant evidence for a causal relationship.

		Without global TH correction				With global TH correction^a^			
Biochemical trait	Cortical trait	$\widehat {GCP}$^b^	*P*^c^	*SE*	*r*_*g*_^d^	$\widehat {GCP}$	*P*	*SE*	*r*_*g*_
C-reactive protein	Lateral occipital TH	0.69	1.15 × 10^–22^	0.16	–0.11	0.60	5.93 × 10^–6^	0.21	–0.05
C-reactive protein	Lingual TH	0.66	4.55 × 10^–54^	0.14	–0.12	0.69	1.41 × 10^–73^	0.19	–0.07
C-reactive protein	Fusiform TH	0.74	6.94 × 10^–24^	0.15	–0.17	0.42	0.28	−0.39	–0.15
Haematocrit %	Insula TH	0.66	5.14 × 10^–15^	0.22	0.12	0.46	0.30	0.34	0.05
RBC count	Insula TH	0.77	2.60 × 10^–26^	0.17	0.12	0.35	0.25	0.35	0.06
Vitamin D	Temporal pole TH	0.61	7.61 × 10^–03^	0.24	0.17	0.72	2.63 × 10^–7^	0.18	0.22

^a^Denotes LCV analyses conducted using GWAS summary statistics produced with mean global cortical TH used as a covariate.

^b^The posterior mean genetic causality proportion.

^c^*P* value which tests whether the $\widehat {GCP}$ estimate is significantly different from zero.

^d^Genetic correlation coefficient.

### Evidence of a causal effect of C-reactive protein on reduced lingual thickness

The total effect of CRP (natural log transformed mg/L) and vitamin D (as 25-hydroxyvitamin D, natural log transformed nmol/L) on regional cortical TH (in mm) was further quantified via two-sample Mendelian randomisation (MR), leveraging instrumental variables (IVs) from non-UKBB cohorts [17, 18]. Six trait pairings were examined (CRP → lingual & lateral occipital TH, vitamin D → temporal pole TH, ± mean TH correction), thus we consider a Bonferroni-adjusted *P*_*IVWm*_ < 0.0083 (0.05/6) statistically significant.

**Fig. 5:**
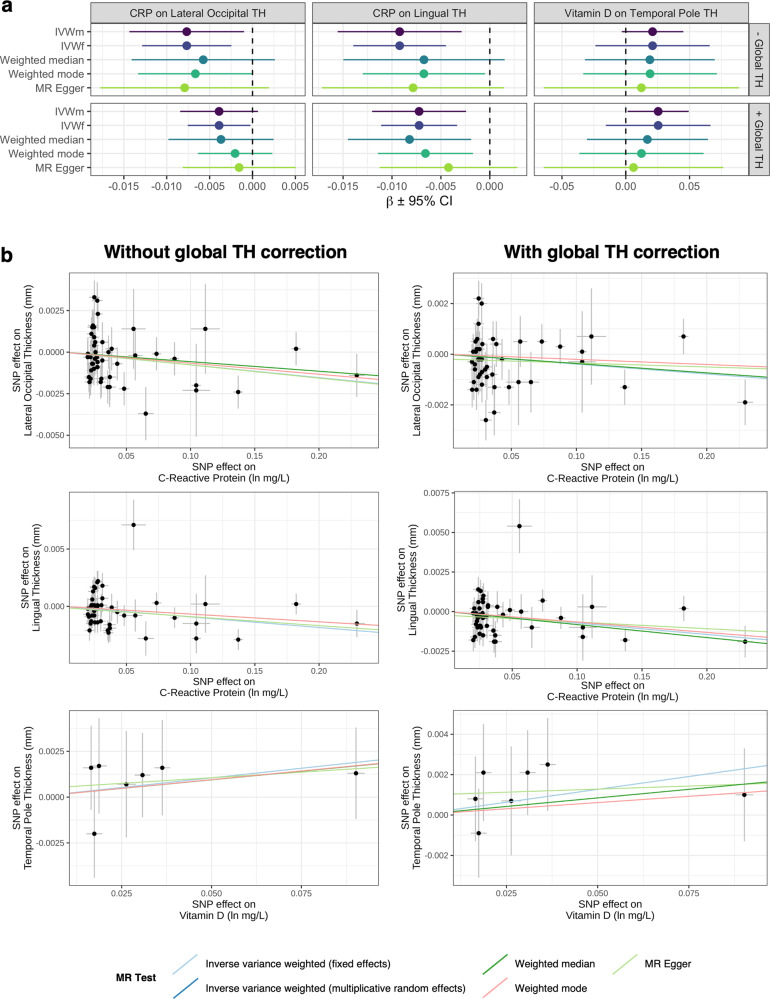
Estimated effect of C-reactive protein and vitamin D exposures on regional cortical thickness measures. **a** Effect size estimates (± 95% CI) of C-reactive protein and vitamin D exposures on cortical TH GWAS before and after covarying for global mean cortical TH. **b** Scatter plots comparing IV effect sizes in exposure and outcome GWAS. Each trendline corresponds to one of five MR methods utilized for sensitivity analysis.

For the CRP analyses, 48 CRP-associated IVs were identified after harmonisation and removal of palindromic SNPs. Interestingly, we identified evidence suggesting each natural log-transformed mg/L increase in CRP was associated with a significant reduction in lingual TH (*β*_*IVWm*_ = –0.009, *SE*_*IVWm*_ = 0.003, *P*_*IVWm*_ = 0.004) after Bonferroni correction, consistent with LDSR and LCV results (Fig. 5a, b, Table S9). A range of sensitivity analyses were subsequently employed to assess the rigor of the IVW_m_ estimate. Using the less conservative IVW estimator with fixed effects (IVW_f_) decreased the standard error as anticipated (*β*_*IVWf*_ = –0.009, *SE*_*IVWf*_ = 0.002, *P*_*IVWf*_ = 1.39 × 10^−4^), while the weighted mode estimator (*β*_*wMode*_ = –0.007, *SE*_*wMode*_ = 0.003, *P*_*wMode*_ = 0.040) also supported the putative effect of CRP on lingual TH (Fig. 5a, b, Table S10). Although the weighted median and MR Egger methods did not reach statistical significance, both causal estimates were directionally consistent (Fig. 5a, b, Table S10). Interestingly, covarying for mean cortical TH further decreased the standard error across all methods, with the weighted median (*β*_*wMedian*_ = –0.008, *SE*_*wMedian*_ = 0.003,*P*_*wMedian*_ = 0.011) estimator attaining statistical significance after including this covariate (Fig. 5a, b, Table S10).

A suggestively significant (*P*_*BH*_ < 0.05) causal association for CRP on lateral occipital TH was additionally uncovered using the IVW_m_ estimator (*β*_*IVWm*_ = –0.008, *SE*_*IVWm*_ = 0.003, *P*_*IVWm*_ = 0.025), however, this was attenuated after covarying for mean cortical TH (*β*_*IWVm*_ = –0.004, *SE*_*IVWm*_ = 0.002, *P*_*IVWm*_ = 0.093; Fig. 5a, b, Table S10). Likewise, the IVW_f_ estimator (*β*_*IVWf*_ = –0.008, *SE*_*IVWf*_ = 0.003, *P*_*IVWf*_ = 0.004) was weakened after correcting for mean TH (*β*_*IVWf*_ = –0.004, *SE*_*IVWf*_ = 0.002, *P*_*IVWf*_ = 0.037; Fig. 5a & b, Table S10). The remaining sensitivity analyses were directionally consistent, albeit insignificant (Fig. 5a, b, Table S10). Across all analyses, we identified no substantial evidence for outlier SNPs using MR-PRESSO and leave-one-out analyses, although the IV exposure-outcome effects exhibited evidence for SNP heterogeneity in both instances (Cochran’s *Q* ≥ 71.84, *P* ≤ 0.01; Tables S11–13). This heterogeneity, however, is not unexpected given the underlying biological complexity of these traits. Moreover, the MR-PRESSO global pleiotropy tests were consistently insignificant (*P* = 1) and the Egger intercepts were not significantly different than zero (*P* ≥ 0.26), providing statistical evidence of no unbalanced pleiotropy (Tables S11 & S13).

We also analysed the effect of natural log-transformed vitamin D concentrations on temporal pole TH using seven harmonised, nonpalindromic IVs from the SUNLIGHT consortium [18]. No causal estimates were statistically significant, apart from the IVW_m_ estimator after covarying for mean cortical TH, which was nominally significant (*β*_*IVWm*_ = 0.025, *SE*_*IVWm*_ = 0.012, *P*_*IVWm*_ = 0.038; Fig. 5a, b, Tables S9 & S10). Similarly, all trait pairings which marginally failed to pass the LCV cut-off or did not survive correction for global TH failed to exhibit reliable evidence for a causal effect (Table S14 & S15).

### Direct causal effect of C-reactive protein observed on lingual thickness conditioned on BMI and IL-6

**Fig. 6:**
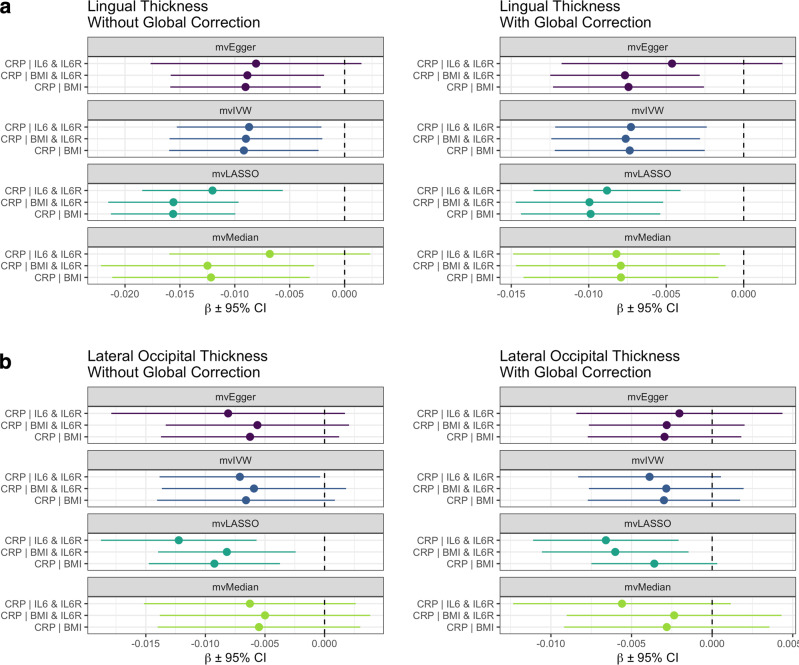
Direct effect of C-reactive protein on lingual and lateral occipital TH conditioned on BMI and IL-6 signalling. Three multivariable MR models were constructed to estimate the direct effect of CRP on lingual (**a**) and lateral occipital (**b**) TH conditioned on body mass index (BMI) and interleukin-6 (IL-6) signalling, specifically: CRP conditioned on IL-6 and its receptor IL-6R, BMI, and IL-6R and BMI. All models were tested using four multivariable MR methods: mvIVW, mvEgger, mvLASSO and mvMedian.

We next utilised multivariable MR (MVMR) to investigate the direct effect of CRP on lingual and lateral occipital TH conditioned on BMI and IL-6 signalling, as these variables are thought to be closely related to circulating CRP levels [19, 20]. Four MVMR methods were implemented, specifically an IVW MVMR estimator with multiplicative random effects, a median-based MVMR estimator, an Egger regression-based MVMR estimator, and a LASSO-type regularisation approach. Overall, CRP exhibited a robust negative relationship with lingual TH when conditioned on BMI and IL-6 signalling, and this was consistent after covarying for mean cortical TH (Fig. 6a, Table S16). For example, we found that the direct effect of CRP conditioned on BMI and IL-6 signalling was similar to the univariate estimates before (*β*_*mvIWVm*_ = –0.009, *SE*_*mvIVWm*_ = 0.004, *P*_*IVWm*_ = 0.011) and after (*β*_*mvIWVm*_ = –0.008, *SE*_*mvIVWm*_ = 0.002, *P*_*mvIVWm*_ = 0.002) covarying for mean cortical TH (Fig. 6a, Table S16). Furthermore, there was limited evidence of an effect of BMI or IL-6 signalling on lingual TH conditioned on CRP (Table S16). In contrast, casual estimates for the direct effect of CRP on lateral occipital TH were generally weak, reflecting findings from univariate MR (Fig. 6b, Table S16). Although some non-zero causal estimates were obtained using mvLASSO models, BMI also exhibited negative causal estimates when conditioned on CRP and IL-6 signalling utilising MVMR IVW and Egger models, suggesting a potential effect on lateral occipital TH (Table S16).

### Limited transcriptome-wide overlap amongst causally linked trait pairings

**Fig. 7:**
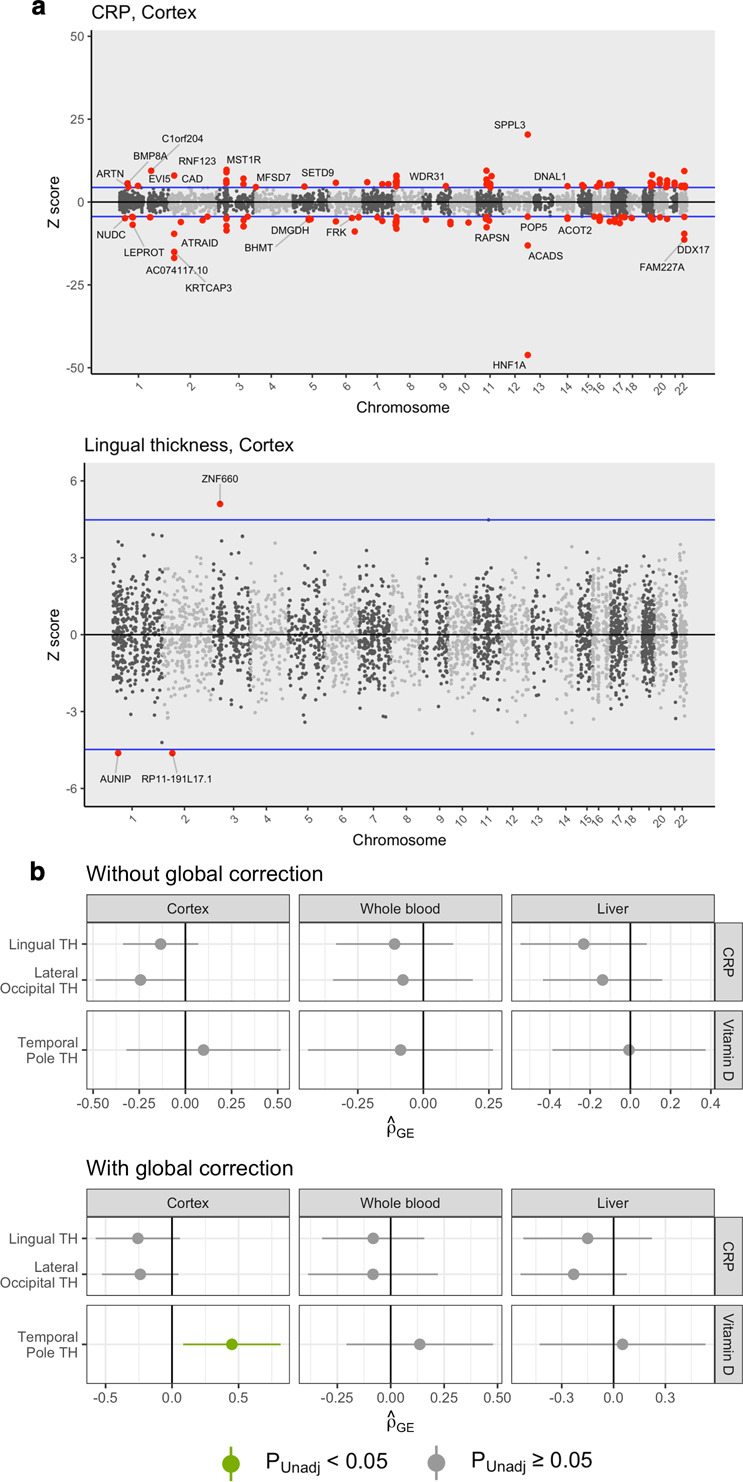
Transcriptome-wide correlation of TWAS *Z-*score profiles between biochemical and cortical traits. **a** Miami plots depicting TWAS results for two representative traits – CRP and lingual TH (without correction for global TH) – using cortical expression weights. Horizontal blue lines correspond to *P*_*Bonferroni*_ < 0.05. **b** Correlation of TWAS *Z-*score profiles amongst all trait pairings with evidence for a causal relationship using RHOGE. Across all tissues, no trait pairings were significantly correlated after multiple testing correction, however the direction of *ρ*_*GE*_ was generally consistent with genetic correlations. Note that temporal pole TH and vitamin D expression profiles were nominally correlated (*ρ*_*GE*_ = 0.45, *P* = 0.023, *SE* = 0.187) after correction for global TH and using cortical weights.

Transcriptome-wide association studies (TWAS) were performed for trait pairings with strong LCV causal estimates to compare genetically predicted gene expression profiles utilising pre-computed SNP-expression weights for cortical tissue, whole blood and liver tissue (see Table S17 for full results). At a *P*_*Bonferroni*_ < 0.05 cut-off, the average number of associated genes per trait across all tissues was as follows: CRP = 157, vitamin D = 37, lateral occipital TH = 1, lingual TH = 4 and temporal pole TH = 2 (see Fig. 7a for representative Miami plots, see Fig. S3 and Table S18 for full results). Given the sparse identification of associated genes for the cortical traits, no overlapping genes were identified (Table S19). However, after correcting for global TH, *RP11-148021.6*, was found to overlap between CRP (*Z*_*TWAS*_ = 6.03) and lateral occipital TH (*Z*_*TWAS*_ = –4.46) using cortical expression weights (Table S18 & S19). Utilising a more-liberal Benjamini-Hochberg significance cut-off, genes overlapping CRP and lingual TH were identified as follows: cortical weights: *KRT18P34, PPFIA1, RP11-555M1.3*, whole blood weights: *KRT18P34, PPFIA1, ZNF660* and liver weights: *PPFIA1* (Tables S18 & S19). Interestingly, *PPFIA1*, which encodes the synaptic scaffolding protein Liprin-α-1 [42], was represented across all three tissues with consistently negative *Z-*scores in the CRP TWAS and positive *Z-*scores in the lingual TH TWAS. Limited transcriptome-wide correlation (*ρ*_*GE*_) was uncovered amongst trait pairings with evidence for causality, however vitamin D and temporal pole TH exhibited nominal positive correlation after correction for global TH using cortical weights (*ρ*_*GE*_ = 0.45, *P* = 0.023, *SE* = 0.187; Fig. 7b, Table S20, Fig. S4). However, we also identified only a limited number of transcriptome-wide associated signals for the cortical properties suggesting that greater sample sizes are required, as well as more tissue and cell-type specific expression weights.

## Discussion

Circulating biochemical factors play a critical role in the development and homeostasis of the central nervous system, however it is unclear whether individual biochemical exposures can modulate structural characteristics of specific brain regions. In the present study, we investigated shared genetic architecture and causal relationships between a panel of blood-based biochemical traits and cortical structure with direct relevance to psychiatric disorders. Our analyses revealed striking genetic overlap between these biochemical traits and measures of cortical SA and TH, in which SA was predominantly correlated with blood cell counts, various nutrients, enzymes, CRP and other biomarkers, whereas TH was largely associated with RBC-related traits and CRP. Interestingly, subsets of these pairings have putative phenotypic correlations in observational studies. For instance, circulating levels of biochemical factors such as triglycerides [43], haemoglobin [44], CRP [9, 45] and glucose [46] have been previously associated with cortical structure in a manner directionally consistent with our findings. Furthermore, the widespread negative correlations between immune-related traits and measures of cortical SA suggest elevated immune activity may correlate with cortical SA deficits, in line with well-established hypotheses regarding the damaging effects of inflammation on cortical integrity during aging [47] and in neurodegenerative disorders [48, 49]. In a more-extreme example, diminished RBC function in sickle cell disease is associated with cortical thinning [50], broadly supporting the positive correlation between RBC-related traits and cortical TH. In conjunction with these results, we uncovered clear divergence between SA and TH measures, in which six (out of seven) biochemical clusters exhibited discordant correlation profiles. Genetic comparison of cortical SA and TH has previously revealed these characteristics are genetically independent [51] and exhibit differing profiles of genetic correlation with respect to neurobehavioral traits and psychiatric disorders [15], implying these structural properties are regulated via independent biological mechanisms. This is consistent with neuroanatomical studies reporting a range of concordant and discordant relationships between region-specific SA and TH with age [52–54] and in the context of psychiatric illness [55, 56]. Similar studies have uncovered dysregulation of intracranial and subcortical volumes in psychiatric disorders [57, 58], however exploratory analysis of these features in the current study (utilising ENIGMA2 summary statistics [59]) failed to identify any correlated trait pairings (Table S21). Although many of the genetic correlations reported in the current study exhibited little genetic evidence for a causal relationship, and are therefore likely indicative of horizontal pleiotropy, these findings are nonetheless important for the identification of genes affecting both cortical structure and biochemical traits. Future repetition of these analyses with larger sample size cortical GWAS may improve the causal estimates obtained in this study and yield further putative relationships with implications for general brain health, as well as psychiatric and neurodegenerative illness.

By utilising genetically informed methods of causal inference, strong evidence for a putative causal relationship was identified for CRP on lingual thinning, while comparatively weaker evidence was obtained for lateral occipital thinning. CRP is widely characterised as an acute inflammatory protein synthesised by hepatocytes as a soluble homopentamer (pCRP) in response to IL-6 and IL-1β signalling [60]. After biosynthesis, pCRP enters systemic circulation and dissociates into proinflammatory monomers (mCRP) at the site of insult, which increase the abundance of M1 macrophages and Th1 T-cells, thereby activating a robust immune response with potentially deleterious effects on host tissues [61]. Persistent elevation of CRP is therefore strongly associated with chronic, low-level inflammation thought to negatively impact the structural integrity of brain regions. For instance, elevated CRP is positively correlated with cortical thinning [9, 45] and decreased grey matter volume [62, 63], while deposition of mCRP has been identified at sites of neurodegeneration [64]. Although CRP-associated thinning of the lingual and lateral occipital regions has not been explicitly reported in previous studies, recent work has shown elevated CRP is associated with lower regional cerebral blood flow to the lingual gyrus, suggesting alteration of cerebral vasculature may contribute to spatially-restricted cortical thinning [65]. Despite this extensive observational evidence, the exact mechanisms through which CRP alters cortical structure remains unclear due to difficulties in dissecting casual relationships via observational studies. Whilst we utilised TWAS to identify common genes amongst CRP, lingual TH and lateral occipital TH to explore shared pathways with potential biological significance, few genes were significantly associated with the cortical measures across all tested tissues, limiting our capacity to genetically probe the mechanistic basis through which CRP modulates cortical integrity in a region-specific manner. This could likely be improved in future studies by boosting the discovery GWAS sample size and specificity of brain regions available, particularly as the FUSION approach is limited to genes with a significantly heritable *cis*-acting component. Moreover, the genetic overlap between these cortical and biochemical traits may not be heavily mediated by *cis*-acting influences on mRNA expression, and thus, integration of other functional data, such as chromatin and methylome-related annotations, could yield more genes associated with these traits. However, we did identify consistent representation of *PPFIA1* across all three tissues, with negative *Z-*scores in the CRP TWAS and positive *Z-*scores in the lingual TH TWAS. This gene encodes Liprin-α-1, a synaptic scaffolding molecule associated with axon targeting, synaptic morphogenesis synaptic vesicle transport and recruitment of AMPA receptors [42], thus genetically mediated regulation of this gene may contribute to the interrelationship between CRP and lingual TH. We additionally note that reverse causality is unlikely for these putative relationships given the strong directional evidence identified via LCV.

Mounting observational evidence suggests psychiatric disorders including schizophrenia, major depressive disorder and bipolar disorder are associated with elevated CRP levels [9, 66, 67]. In the case of schizophrenia, high peripheral CRP is correlated with negative symptoms [68] as well as cortical thinning in frontal, insula and temporal regions [9]. However, no studies to date have specifically reported CRP-associated thinning of the lingual and lateral occipital regions, nor CRP-associated alteration of their accompanying cognitive functions, such as visual memory (lingual), visual imagery (lingual) and object recognition (lateral occipital) [69, 70]. Thus, it is unclear whether the putative causal relationships identified in the current study bear direct functional significance for psychiatric disorders. More-generally, conflicting results from MR and LCV analyses have recently reported a protective effect for CRP in schizophrenia, obsessive-compulsive disorder and anorexia nervosa, and a causal effect in major depressive disorder [13, 71], further obscuring the underlying role of this protein in psychiatric illness. While these discoveries seemingly conflict with our results and previous observational evidence, complex dimensions of CRP function may underpin these divergent findings. Specifically, recent evidence suggests pCRP can dampen inflammation by skewing macrophages and T-cells towards anti-inflammatory M2 and Th2 phenotypes [61], respectively, suggesting that while high serum pCRP may be indicative of a pervasive acute phase inflammatory response, moderately elevated pCRP may indeed exert a protective effect against underlying chronic inflammation. This is an issue for observational inferences, which employ tests such as the mean difference in CRP between cases and controls, as the magnitude of CRP elevation can often be relatively small and more indicative of this moderate state. Regardless of the underlying mechanism, these findings collectively advocate further investigation of the direct and indirect effects of CRP on neuronal integrity to further reconcile the impact of CRP levels on cortical structure and psychiatric disorders.

We additionally identified a positive causal relationship for vitamin D on temporal pole TH via LCV, however, these findings were not supported by MR. Furthermore, a nominally significant transcriptome-wide correlation between these traits was observed after correcting for global TH. While evidence for a causal relationship between vitamin D levels and temporal pole TH could not be conclusively established, our findings are broadly consistent with a recent cross-sectional study wherein total vitamin D intake and vitamin D supplementation were specifically correlated with enhanced temporal lobe TH in cognitively normal, older adults (>65 years) [72]. Similar studies have previously reported increased prefrontal and cingulate TH in individuals with higher serum vitamin D concentrations [73, 74], suggesting any correlation between vitamin D and cortical TH may not be spatially restricted to structures such as the temporal pole. Indeed, vitamin D receptors and other vitamin D-associated genes (such as *CYP27B1*) are expressed throughout the brain [75], while functional studies indicate vitamin D promotes axon outgrowth [76], enhances the expression of neurotrophic factors (such as NGF) [76], and exhibits neuroprotective effects [77], implying a particularly important role throughout the brain. Although a causal effect of vitamin D on brain structure is yet to be definitively established, a potential relationship with the temporal pole would nonetheless be significant for general cognition and psychiatric illness given the diverse range of functions attributed to this region, including visual cognition, face recognition, visual memory, visual discrimination, social cognition, language and semantic processing and autobiographic memory, amongst others [78, 79]. Subsets of these cognitive functions are indeed disrupted in schizophrenia and other psychiatric disorders [80–82], and furthermore, structural alterations affecting the temporal pole have also been reported in schizophrenia [83], obsessive-compulsive disorder [84], bipolar disorder [85], major depressive disorder [86] and attention deficit hyperactive disorder [87]. In conjunction with these findings, low neonatal and adult vitamin D levels have been previously identified as a risk factor for schizophrenia [88, 89], however, it is unclear whether low vitamin D directly contributes to schizophrenia-associated alterations to cortical structures such as the temporal pole, thus ongoing studies will prove critical in determining whether vitamin D impacts cortical integrity, particularly in a manner relevant to psychiatric illness.

In summary, our findings suggest subsets of biochemical exposures and cortical structural properties share genetic architecture and, in some cases, exhibit evidence for causal relationships. We acknowledge several limitations and caveats with respect to interpretation of the presented data. Firstly, all analyses in the current study are inherently subject to limitations and biases potentially associated with the utilised summary statistics, such as population stratification [90] and selection bias [91]. The UKBB cohort in particular is composed of individuals over the age of 40, thus variants associated with developmentally sensitive modulation of the biochemical traits could not be examined. Utilisation of genetic data from younger individuals in future studies may yield further insights at developmentally informative timepoints. We also note that while our MVMR models accounted for known factors associated with CRP levels (i.e. IL-6, IL-6R and BMI), our selection of potential confounders was not exhaustive. Another important consideration is the regional specificity of our findings. We assert that region-specific trait pairings which survived correction for global measures in the LCV and MR analyses are particularly robust, since biological mechanisms underpinning global and regional cortical structure are likely to overlap extensively, thus correcting for global measures may eliminate a large proportion of the genetic signal foundational to region-specific structure. Global corrections were therefore considered excessively conservative for the discovery-oriented genetic correlation analyses, however the partitioning of global correlations into discrete region-specific profiles suggests uncorrected regional summary statistics are nonetheless highly informative. Finally, we caution that all reported causal relationships require validation in randomised controlled trials to confirm the putative causal effects. Despite the limitations of these analyses, genetically informed causal inference represents an exciting opportunity to screen and prioritise biochemical traits *en masse* to guide future investigation of these exposures in the context of neuronal function, brain cytoarchitecture and psychiatric illness.

## Supplementary information

Supplementary Figures

Supplementary Tables

## Data Availability

All code utilised in the current study are available from the authors on request.
